# Clinical Implication of the Relationship between Antimicrobial Resistance and Infection Control Activities in Japanese Hospitals: A Principal Component Analysis-Based Cluster Analysis

**DOI:** 10.3390/antibiotics11020229

**Published:** 2022-02-10

**Authors:** Tomokazu Shoji, Natsu Sato, Haruhisa Fukuda, Yuichi Muraki, Keishi Kawata, Manabu Akazawa

**Affiliations:** 1 Department of Public Health and Epidemiology, Meiji Pharmaceutical University, Noshio, Kiyose 204-8588, Tokyo, Japan; tshohji@yamanashi.ac.jp (T.S.); y161154@std.my-pharm.ac.jp (N.S.); 2 Department of Pharmacy, University of Yamanashi Hospital, Shimokato, Chuo 409-3898, Yamanashi, Japan; kkawata@yamanashi.ac.jp; 3 Department of Health Care Administration and Management, Graduate School of Medical Sciences Kyushu University, Maidashi, Higashi, Fukuoka 812-8582, Japan; h_fukuda@hcam.med.kyushu-u.ac.jp; 4 Department of Clinical Pharmacoepidemiology, Kyoto Pharmaceutical University, Yamashina, Kyoto 607-8414, Japan; y-muraki@mb.kyoto-phu.ac.jp

**Keywords:** antimicrobial resistance, infection prevention and control, antimicrobial stewardship, hospital, cluster analysis, principal component analysis

## Abstract

There are few multicenter investigations regarding the relationship between antimicrobial resistance (AMR) and infection-control activities in Japanese hospitals. Hence, we aimed to identify Japanese hospital subgroups based on facility characteristics and infection-control activities. Moreover, we evaluated the relationship between AMR and hospital subgroups. We conducted a cross-sectional study using administrative claims data and antimicrobial susceptibility data in 124 hospitals from April 2016 to March 2017. Hospitals were classified using cluster analysis based the principal component analysis-transformed data. We assessed the relationship between each cluster and AMR using analysis of variance. Ten variables were selected and transformed into four principal components, and five clusters were identified. Cluster 5 had high infection control activity. Cluster 2 had partially lower activity of infection control than the other clusters. Clusters 3 and 4 had a higher rate of surgeries than Cluster 1. The methicillin-resistant *Staphylococcus aureus* (MRSA)/*S. aureus* detection rate was lowest in Cluster 1, followed, respectively, by Clusters 5, 2, 4, and 3. The MRSA/*S. aureus* detection rate differed significantly between Clusters 4 and 5 (*p* = 0.0046). Our findings suggest that aggressive examination practices are associated with low AMR whereas surgeries, an infection risk factor, are associated with high AMR.

## 1. Introduction

Antimicrobial resistance (AMR) is an emerging global public health crisis. In Japan, the National Action Plan on AMR 2016–2020 issued in 2016, demanded medical institutions to promote comprehensive AMR control, linking the efforts of the existing infection control team (ICT) at the field level with those of the antimicrobial stewardship program (ASP) [1]. Methicillin-resistant *Staphylococcus aureus* (MRSA) is the most common AMR and should be monitored by each institution [2]. One of the goals of the action plan was to reduce the MRSA resistance rate to 20% by 2020 [1]. In 2020, the MRSA resistance rate decreased; however, it is still much higher than the corresponding outcome indices [3].

Quality indicators (QIs) related to infection control and the proper use of antibacterial agents including hand disinfection compliance rates, implementation of facility guidelines [4] and the implementation rate of Therapeutic Drug Monitoring (TDM), have been proposed [5]. In Japan, a surveillance system called Japan Surveillance for Infection Prevention and Healthcare Epidemiology (J-SIPHE) is in operation, and clinical indicators (e.g., antibacterial drug usage, blood culture collection rate, etc.) of participating institutions were collected [6]. In this way, various Qis have been proposed as infection control indicators, but implementing them remains difficult as there is no clear benchmark. Individual indicators are intricately intertwined and should be evaluated comprehensively [7].

The Japanese Ministry of Health, Labour and Welfare (MHLW) incorporated the medical fee for infection prevention and control (IPC), which is classified into two types (IPC type 1 or IPC type 2) [8]. Type 1 applies to physicians or nurses with >0.8 full-time equivalent (FTE), while type 2 applies to each member with >0.5 FTE [9,10]. However, some facilities had difficulties claiming the IPC medical fee because of shortages in infection control supply materials, as well as human resources and facility equipment [11,12]. Therefore, we believe that it is necessary to evaluate not only IPC and ASP but also the status of the facility when considering the relationship with AMR. 

The optimal Qis for evaluating IPC are unclear. Additionally, there are differences in medical care, human resources, and physical resources even in facilities with a similar type of IPC medical fee. Thus, it is difficult to distinguish between facilities based on specific QI or medical fee. A more practical approach to understanding individual IPC and its underlying mechanism is to consider the specific facility factors and infection control measures. Furthermore, there is a need to evaluate the factors that may be related to AMR.

**Figure 1:**
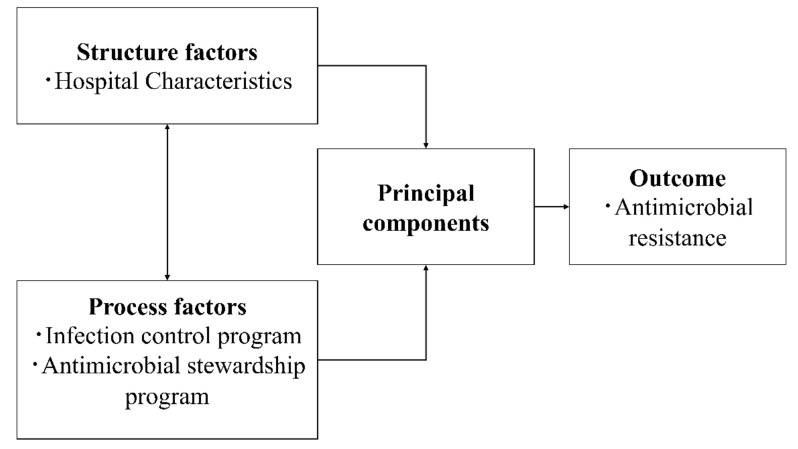
Conceptual framework.

In this study, we aimed to objectively summarize the variables associated with infection control and identify facility clusters based on structure and process factors. Moreover, we assessed the relationships between AMR and these clusters to clarify the factors that may affect AMR. The conceptual framework that guided our study was the quality of care model developed by Donabedian [13]. This conceptual framework comprises the following three domains: structure factors, process factors, and AMR (Figure 1). We conducted a principal component analysis to transform the selected variables included in the cluster analysis [14].

## 2. Results

A total of 124 hospitals were analyzed, excluding 21 hospitals from 145 participating hospitals in this study. Data from these 21 hospitals were missing the following variables: the diagnostic procedure combination/per-diem payment system (DPC/PDPS) data file (4), the Japan Nosocomial Infections Surveillance (JANIS) specimen data (*n* = 10), JANIS patient age data (*n* = 6), and JANIS microbial susceptibility data (*n* = 1). Characteristics of the 124 analyzed hospitals are described in Table 1. The structure factors represented by the median number of beds in the facilities was 330 (interquartile range [IQR]: 223–478) beds; the median length of hospital stay was 12.4 (IQR: 11.1–14.1) days; the rate of surgeries for surgical site infection (SSI) surveillance among all surgeries was 26.5% ± 5.6%; 59.7% of the facilities were located in the eastern region of Japan; and 92.7% claimed IPC type 1 medical fee. The process-related factors were represented by the TDM implementation rate for vancomycin (79.2%), multiple sets of blood culture (81.1%), blood culture contamination (3.1%), number of Clostridioides difficile (CD) detection tests (4.2/1000 bed days), blood culture collected prior to broad spectrum antibiotic therapy (60.1%), and the number of bacterial tests (9.8/100 bed days). The median MRSA/Staphylococcus aureus (S. aureus) detection rate was 42.3% (IQR: 33.3–52.5).

We performed a principal component analysis based on an optimal subset of ten selected variables (in the first principal component created with all variables, there were no variables with eigenvectors >0.4, when eleven variables were selected, the cumulative eigen-value was less than 60%). Four structure factors including average length of stay, rate of surgeries, region, and IPC type were selected. By contrast, six process factors including the TDM implementation rate for vancomycin, multiple sets of blood cultures, blood culture contamination, the number of bacterial tests, the number of CD-detected tests, and blood culture collected prior to broad spectrum antibiotic therapy were selected. The first four principal components with eigenvalues >1 explained 63.0% of the variance and were therefore retained for further cluster analysis (Table A1). The first principal component accounted for 24.9% of variance for which the three factor scores with the largest eigenvectors were the number of bacterial tests, blood culture collected prior to broad spectrum antibiotic therapy, and number of CD detected tests. Therefore, the first principal component was characterized as the component for “bacterial test”. Likewise, the second principal component, accounting for 14.8% of variance, was characterized as “surgeries”, because the components were the rate of surgery, average length of stay, and region. The third principal component, which accounted for 12.9% of variance, was characterized as the “ability of infection control team”, as the components were the TDM implementation rate for vancomycin, medical fee for IPC type, and rate of blood culture contamination. The fourth principal component, which accounted for 10.8% of variance, was characterized as “skill in performing blood cultures”, as the components were multiple sets of blood cultures performed and blood culture contamination.

**Figure 2:**
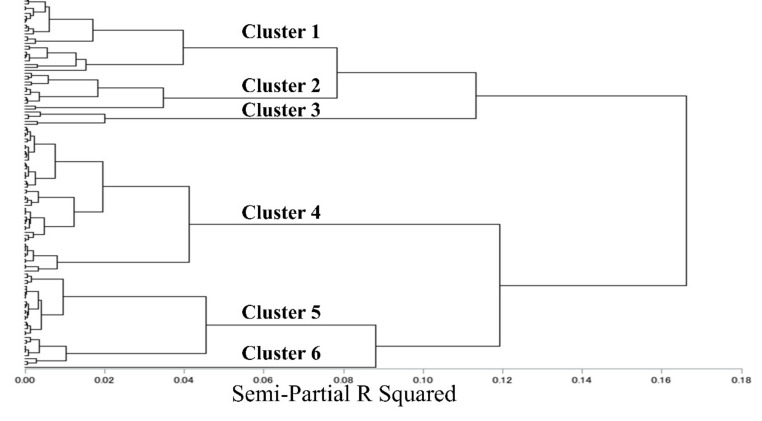
Dendrogram illustrating the results of the cluster analysis. Cluster 1 (*n* = 25), Cluster 2 (*n* = 13), Cluster 3 (*n* = 5), Cluster 4 (*n* = 49), Cluster 5 (*n* = 31), Cluster 6 (*n* = 1). Cluster 6 only had one facility, thus, it was considered an outlier and was excluded in the subsequent analyses.

A hierarchical cluster analysis was performed among the 124 facilities based on the four principal components derived from the principal component analysis, and six distinct clusters were obtained (Figure 2). Cluster 6 included only one facility with data on the number of bacterial tests (52.0/100 bed days) and the number of CD detected tests (27.0/1000 bed days), and those values were considered outliers because they were significantly different from those of the other clusters. Therefore, we excluded this Cluster from further analysis. Cluster 1 (*n* = 25; 20%), Cluster 2 (*n* = 13; 11%), Cluster 3 (*n* = 5; 4%), Cluster 4 (*n* = 49; 40%), and Cluster 5 (*n* = 31; 25%) were established.

Table 2 shows the clinical characteristics of the five clusters. The median number of beds decreased from the highest to lowest, as follows: Cluster 5, Cluster 4, Cluster 2, Cluster 1, and Cluster 3. Cluster 2, Cluster 4, and Cluster 5 included almost all facilities with IPC type 1. Cluster 5 had the shortest average length of stay (10.9 days) and the highest number of bacterial tests (14.6/100 bed days). Cluster 4 had the highest rate of surgeries (29.3%) and second longest average length of stay (13.2 days). Additionally, Cluster 4 had a lower number of blood cultures collected prior to broad spectrum antibiotic therapy (59.2%) and a lower number of bacterial tests (8.5/100 bed days) than Cluster 5. All facilities in Cluster 2 claimed IPC type 1. Meanwhile, Cluster 2 had the lowest number of multiple sets of blood cultures (46.3%) and the highest rate of contaminated blood cultures (4.1%). Cluster 1 was composed of facilities with both IPC types 1 and 2 and the lowest rate of surgeries (22.1%), while the number of blood cultures collected prior to broad spectrum antibiotic therapy (40.6%) was lower than for Cluster 2 and Cluster 5. Cluster 1 also had the second lowest number of bacterial tests (6.8/100 bed days). Cluster 3 was composed of facilities with IPC type 2 only. The average length of stay in Cluster 3 was longer than that in other clusters (14.9 days), and it had the lowest TDM implementation rate for vancomycin (9.7%), the lowest rate for blood culture collected prior to broad spectrum antibiotic therapy (30.1%), and the lowest number of bacterial tests (6.7/100 bed days). Table A2 shows more detailed data.

**Figure 3:**
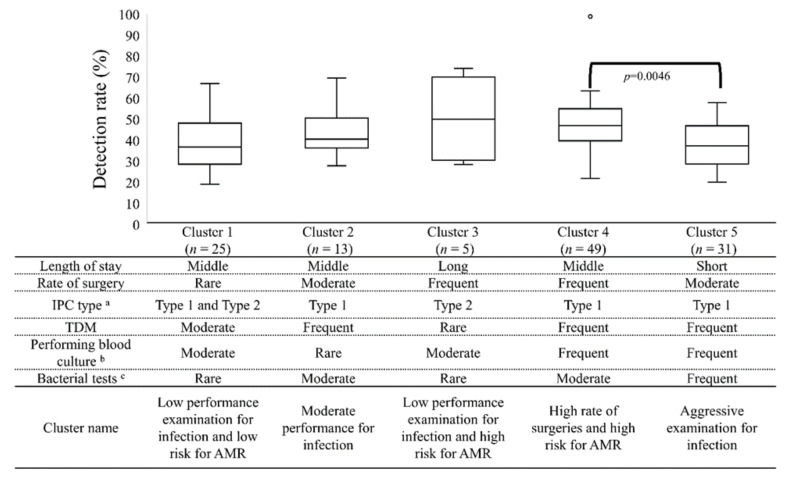
MRSA/*S. aureus* detection rate and summary of cluster characteristics. Each box plot is composed of five horizontal lines that display the minimum and maximum values and the 25th, 50th, and 75th percentiles of the corresponding variable. The cluster summary was categorized into three levels (Frequently, Moderate, Rare) or (Long, Middle, Short) based on the median value at hospital baseline (Table 1) and the comparison of that variable with each cluster (Table 2). ^a^ Medical fee for IPC type 1 or type 2. ^b^ Taking into account multiple sets of blood cultures and contamination of blood cultures. ^c^ Taking into account the number of CD detected test, blood cultures collected prior to broad spectrum antibiotic therapy, and number of bacterial tests. *S. aureus*, *Staphylococcus aureus*; MRSA, methicillin-resistant *S. aureus*; IPC, Infection Prevention and Control; TDM, Therapeutic Drug Monitoring.

Figure 3 shows a summary of the characteristics of the five clusters (based on Table 1 and Table 2) in addition to the relationship between clusters and the MRSA/*S. aureus* detection rate. The MRSA/*S. aureus* detection rate ranked from the lowest to highest as follows: Cluster 1, Cluster 5, Cluster 2, Cluster 4, and Cluster 3 (36.8%, 37.3%, 40.6%, 47.0%, and 50.0%, respectively). The Kruskal–Wallis test followed by the Steel–Dwass test revealed significant differences between Cluster 4 and Cluster 5 (*p* = 0.0046).

## 3. Discussion

In this study, we implemented a principal component analysis-based cluster analysis of structure and process factors, which eventually resulted in the identification of five clusters. Our results indicate that when evaluating the MRSA detection rate for each facility, consideration should be paid to not only process factors but also structure factors including resources (e.g., number of medical staff) or risk factors (e.g., surgery), as these are also related with AMR. 

We conducted multicenter analyses by principal component analysis-based cluster analysis. This method required many samples, which were not only useful in reducing dimensionality but also in detecting the key features of the data. This method has been applied to common diseases, such as chronic obstructive pulmonary disease, dermatomyositis, and chronic heart failure [15,16,17,18]. However, it has scarcely been used in evaluating infection control in health facilities.

This study showed the need to consider AMR in terms of infection control and facility characteristics, especially the number of bacterial examinations and surgeries related to SSI. For instance, the MRSA/*S. aureus* ratio between Cluster 4 and Cluster 5 had significant differences. In this study, Cluster 5 had the highest rate of activity for infection control. International guidelines demonstrate the detection of bacterial species, followed by optimization of antimicrobial stewardship, and prevention of growing AMR [19]. In our study, Cluster 4 had the highest rate of surgeries. The National Healthcare Safety Network reported that *S. aureus* is the most common pathogen of SSIs and that the proportion of SSIs caused by *S. aureus* increased to 30%, with MRSA comprising 49.2% [20]. Many surgeries for SSI surveillance may have reflected the high detection rate of AMR. Cluster 2 showed a higher MRSA/*S. aureus* detection rate than Cluster 5. A previous descriptive study reported by a Japanese university hospital showed that ICT recommendation improved the ratio of multiple sets of blood culture, similarly, affecting AMR [21]. Partial infection control activities may be insufficient (e.g., education of blood culture for medical staff) and may represent poor ICT activity, thereby affecting AMR. Cluster 3 was composed of facilities collecting IPC type 2 medical fees. These facilities did not have enough human resources, medical resources, and stratified facilities, as reported in previous studies [9,10]. In this study, the MRSA/*S. aureus* rate was the highest in Cluster 3, which suggests that IPC type 2 facilities require national level support for better implementation of infection control. Cluster 1 had low rates of infection control activities, surgeries, and MRSA/*S. aureus* detection. A recent retrospective study of one small-sized and eleven medium-sized hospitals (range: 73–354 number of beds) reported that these hospitals had a low number of bacterial tests, which meant that patients with serious infections were likely to be transferred to an acute care hospital. Therefore, AMR-isolation rates were reflected as lower in these hospitals [22]. Cluster 1 may reflect only a few incidents of encountering AMR, and infection-control activity could also possibly be low.

Nevertheless, there are also several limitations to our study. First, various factors that may affect resource use were not considered in our study because of the lack of available data. For example, a previous study reported that compliance with hand hygiene reduced nosocomial infections and MRSA transmission [23]. However, the usage of alcohol disinfection in the hospital is not recorded in the administrative data, and hence could not be used. These data may influence the MRSA/*S. aureus* detection rate. Second, as participation in this study was voluntary, the participating hospitals may not be representative of all hospitals in Japan. Moreover, a project managed by Kyushu University believes that the hospitals that participated in JANIS and participated in this study were already highly conscious of infection control; however, we were not able to evaluate associated biases. Future studies should evaluate not only the recruited hospitals but also all hospitals in Japan. Third, our two sets of data were not linked by individual patient identification. This limitation meant that patients in the DPC data cannot be linked with the JANIS data. Therefore, it was not possible to directly evaluate the medical procedures of patients with submitted bacterial tests. As an alternative, facility-level variables were obtained from the data to achieve our purpose. Our conceptual model showed that AMR is affected by the activity of the facilities; hence, we found the evaluation of variables at the facility-level to be appropriate. In the future, more detailed analyses may be possible if databases containing the provided medical care data and detected bacterial data at the individual patient-level are constructed.

## 4. Materials and Methods

### 4.1. Data Source

The administrative claims data were based on the DPC/PDPS, which is a national administrative claims database for acute inpatient care in Japan. Nationally uniform electronic DPC data include facility information (e.g., number of beds), patient clinical information (e.g., age, sex, disease, and admission and discharge date), and information on medical procedures (e.g., drug administration, surgery, examination, procedure, their codes, and cost) [24,25] used for patient classification and the DPC-based reimbursement system. They are used to improve systems and policies, including hospital management and service reimbursement. The JANIS data, which include microbiological data, provide information regarding patient demographics, specimen reception dates, specimen sources, types of bacteria, and susceptibility test results [26]. Moreover, the JANIS data encompass all testing results regardless of characteristics, such as infection status, colonization, or carrier status. JANIS has been launched as a voluntary AMR surveillance funded by the MHLW and managed by the National Institute of Infectious Diseases. JANIS clinical laboratory division collects comprehensive specimen-based data, which comply with JANIS data format, from participating hospitals each month.

We conducted a cross-sectional database analysis. We used both administrative claims data and microbiological data obtained from a research project managed by Kyu-shu University [27]. The project aimed to analyze the association between infection control, including antimicrobial consumption, and AMR. Kyushu University collected two sets of data from 145 acute care Japanese hospitals, which responded to the call for voluntary study participation.

The DPC/PDPS data and the JANIS data have different datasets. These data have common facility identifications, but not common patient identifications. Furthermore, our research objective was at the facility-level and not at the patient-level. Therefore, the variables collected and calculated were based on the identification of each facility.

### 4.2. Study Inclusion and Exclusion Criteria

This study included patients who had been admitted and included in the DPC data in each facility as well as those with specimen reception dates as indicated in the JANIS data between 1 April 2016, and 31 March 2017. Facilities without records of patient characteristics, hospitalizations, procedures, drugs, surgeries, or microbial susceptibility data were excluded because these variables were necessary for the principal component analysis and cluster analysis.

### 4.3. Variable Definitions and Facility Categories

Variables from the DPC data and JANIS data were obtained, calculated, and summarized at the facility-level and were evaluated as potential candidate variables for the following three domains: structure factor, process factor, and outcome. These definitions are presented in Table A3. The region and number of hospital beds were obtained from the DPC data. The following variables were also calculated from the DPC data: number of patient admissions per year, average length of stay, rate of surgeries, intensive care unit patient admissions, central venous catheter use, urinary catheter use, medical fee for IPC type (type 1 or type 2), hospital status (teaching or non-teaching), hospital charge index (7:1 or 10:1), and pharmaceutical services (claimed or non-claimed). Process factors included the following variables calculated from the JANIS data: multiple sets of blood cultures and contamination of blood cultures. Moreover, the following variables were calculated from DPC data: TDM implementation rate for vancomycin, number of CD detected tests, blood culture collected prior to broad spectrum antibiotic therapy, specimens for culture prior to broad spectrum antibiotic therapy, number of bacterial tests, antimicrobial use density (AUD) of antibiotic injections, and days of therapy (DOT) of antibiotic injections. AMR included the MRSA/*S. aureus* detection rate calculated from the JANIS data.

### 4.4. Principal Component Analysis and Cluster Analysis

Principal component analysis is a dimension-reducing method that replaces the variables in a dataset with a smaller number of derived variables. Dimension reduction may help to remove redundant variables that could possibly hinder the clustering process [18,28]. The goal of the principal component analysis was to extract a number of principal components to be used as clustering variables. One way to perform principal component analysis is by selecting a subset of variables that best approximates all the variables [29]. During variable selection for principal component analysis, we performed the following steps: first, we selected variables that can be considered resources for hospital infection control and excluded similar variables that reflect the size of the hospitals. In this study, medical fee for IPC type had been selected. The AMR domain was also selected for the outcome measure. Second, for the independent variables, correlations between survey variables were computed. Variables that were highly correlated (***γ*** > 0.70) were avoided for simultaneous use in the principal component analysis and only either one was used. The coefficient of variation (CV) was calculated for highly correlated variables, and those with the largest CVs were selected for principal component analysis. Third, the optimal variable subset was explored in performing principal component analysis following these criteria: (1) greater number of variables, (2) fewer number of principal components with an eigenvalue >1, and (3) cumulative eigenvalue >60%. Variables with eigenvectors >0.4 were considered principal components. 

A hierarchical cluster analysis was performed based on the Euclidean distances among the principal components [30]. We implemented the Ward method to minimize the total variance within clusters [31]. The number of clusters were based on the distribution of variables among the clusters.

### 4.5. Statistical Analyses

After performing the cluster analysis and choosing the number of clusters, each variable was compared among the clusters, and each cluster was labeled. We then compared the outcome measure (MRSA/*S. aureus* detection rate), which was the parameter that had not been used for the principal component analysis and cluster analysis. Continuous variables are expressed as means (standard deviation [SD]) or medians (interquartile range [IQR]) depending on whether their distributions are normal or skewed. Categorical variables are expressed as numbers (percentages) and binary variables are coded as 0/1 forms. Differences in characteristics between the clusters were assessed using an analysis of variance for continuous normally distributed variables, and non-parametric Kruskal–Wallis tests were used for non-normally distributed variables. All statistical analyses were performed with SAS 9.4 (SAS Institute, Cary, NC, USA), and *p* < 0.05 was defined as statistically significant.

## 5. Conclusions

We established a novel exploratory statistical methodology for multicenter Japanese hospitals, which led to the identification of subgroups based on structure and infection control factors. Our results suggest the importance of a multidimensional assessment of AMR, involving structure and infection control factors. Antimicrobial stewardship, including aggressive examination, is associated with low AMR while surgeries, an infection risk factor, are associated with high AMR. For more detailed research, it would be desirable to establish a database associating patient information with microbiological detection.

## Figures and Tables

**Table 1:** Hospital baseline characteristics for principal component analysis: median (interquartile range) or %.

Characteristics	Hospital Baseline (*n* = 124)
**Structure factors**	
Number of beds (beds) ^a^	330 (223–478)
Number of patients admissions per year (patients) ^a^	7099 (4576–11284)
Average length of stay (days)	12.4 (11.1–14.1)
Rate of surgeries (%) ^b^	26.5 (5.6)
ICU patient admissions (%) ^a^	0.3 (0–4.9)
CVC use patients (%) ^a^	6.0 (4.6–8.5)
UC use patients (%) ^a^	12.2 (9.4–15.1)
Region (East Japan: %)	59.7
Medical fee for IPC type 1 (%)	92.7
Teaching Hospital (%) ^a^	87.1
7:1 hospital charge index (%) ^a^	96.0
Pharmaceutical service (%) ^a^	65.3
**Process factors**	
TDM implementation rate for vancomycin (%)	79.2 (67.0–84.9)
Multiple sets of blood cultures (%)	81.1 (68.7–88.5)
Contamination of blood cultures (%)	3.1 (1.9–4.6)
Number of CD detected test (/1000 bed days)	4.2 (2.6–5.4)
Blood culture collected prior to broad spectrum antibiotic therapy (%) ^c^	60.1 (40.8–71.5)
Specimens for culture prior to broad spectrum antibiotic therapy (%) ^a,c^	82.4 (72.6–88.4)
Number of bacterial tests (/100 bed days)	9.8 (6.7–12.9)
AUD of antibiotic injection (/100 bed days) ^a^	15.8 (12.8–19.1)
DOT of antibiotic injection (/100 bed days) ^a^	26.3 (22.4–30.3)
**Antimicrobial resistance**	
MRSA/*S. aureus* detection rate ^a^	42.3 (33.3–52.5)

ICU, Intensive care unit; CVC, Central venous catheter; UC, Urinary catheter; IPC, Infection Prevention and Control; TDM, Therapeutic Drug Monitoring; CD, *Clostridioides difficile*; AUD, Antimicrobial Use Density; DOT, Days of Therapy; MRSA, Methicillin-resistant *Staphylococcus aureus*. ^a^ Variables not used for the principal component analysis. ^b^ Values are expressed as mean (SD, standard deviation). ^c^ Highly correlated was computed (γ = 0.76). Correlation of variation (CV) was calculated for both blood culture collected prior to broad spectrum antibiotic therapy (CV = 34.3) and specimens for culture prior to broad spectrum antibiotic therapy (CV = 18.2); the former was used.

**Table 2:** Hospital characteristics according to the five clusters identified using principal component analysis-based cluster analysis: median or %.

	Cluster 1 (*n* = 25)	Cluster 2 (*n* = 13)	Cluster 3 (*n* = 5)	Cluster 4 (*n* = 49)	Cluster 5 (*n* = 31)	Overall *p*-Value
**Structure factors**						
Number of beds (beds)	269	275	106 ^e^	304 ^e^	466 ^c,d^	0.0002
Number of patients admissions per year (patients)	6303	5789 ^c^	2587 ^b,d,e^	6184 ^c^	11,046 ^c,d^	<0.001
Average length of stay (days)	12.5	13.4	14.9 ^e^	13.2 ^e^	10.9 ^c, d^	<0.001
Rate of surgeries (%) ^f^	22.1 ± 4.8	25.3 ± 4.7	29.1 ± 6.3	29.3 ± 5.4 ^a,e^	25.3 ± 3.7 ^d^	0.0055 ^g^
ICU patient admissions (%)	3.0	0	0	0	3.7	0.0365
CVC use patients (%)	6.1	8.0 ^c^	3.1 ^b,e^	5.6	6.4 ^c^	0.0159
UC use patients (%)	13.3	12.3	14.2	12.1	12.0	0.3219
Region (East Japan: %)	92.0	84.6	0	36.7	70.9	
Medical fee for IPC type 1 (%)	84.0	100	0	100	100	
Teaching Hospital (%)	76.0	92.3	60.0	87.8	96.7	
7:1 hospital charge index (%)	92.0	100	100	94.0	100	
Pharmaceutical service (%)	64.0	76.9	40.0	65.3	64.5	
**Process factors**						
TDM implementation rate for vancomycin (%)	64.3 ^b,d,e^	82.1 ^a^	9.7 ^d,e^	80.0 ^a,c^	80.8 ^a^^,c^	<0.001
Multiple sets of blood cultures (%)	80.6 ^b^	46.3 ^a,c,d,e^	78.0 ^b^	82.3 ^b^	85.0 ^b^	<0.001
Contamination of blood cultures (%)	4.0	4.1	3.5	3.0	2.4	0.0988
Number of CD detected test (/1000 bed days)	3.8	3.7	4.4	3.8 ^e^	5.4 ^d^	0.0114
Blood culture collected prior to broad spectrum antibiotic therapy (%)	40.6 ^d,e^	60.9	30.1 ^e^	59.2 ^a,e^	72.3 ^a,c,d^	<0.001
Specimens for culture prior to broad spectrum antibiotic therapy (%)	72.8 ^d,e^	87.3	63.0 ^e^	81.8 ^a,e^	85.9	<0.001
Number of bacterial tests (/100 bed days)	6.8 ^e^	10.3 ^e^	6.7 ^e^	8.5 ^e^	14.6 ^a,b,c,d^	<0.001
AUD of antibiotic injection (/100 bed days)	15.4 ^e^	18.3	12.8	14.5 ^e^	19.1 ^a,d^	<0.001
DOT of antibiotic injection (/100 bed days)	25.6 ^e^	28.3 ^d^	24.9	23.5 ^b,e^	30.1 ^a,d^	<0.001

ICU, Intensive care unit; CVC, Central venous catheter; UC, Urinary catheter; IPC, Infection Prevention and Control; TDM, Therapeutic Drug Monitoring; CD, Clostridioides difficile; AUD, Antimicrobial Use Density; DOT, Days of Therapy; MRSA, Methicillin-resistant *Staphylococcus aureus*. ^a^ different form Cluster 1 (*p* < 0.05), ^b^ different form Cluster 2 (*p* < 0.05), ^c^ different form Cluster 3 (*p* < 0.05), ^d^ different form Cluster 4 (*p* < 0.05), ^e^ different form Cluster 5 (*p* < 0.05). ^f^ mean ± SD. ^g^ ANOVA/Tukey–Kramer test.

## Data Availability

The data are not publicly available as the participants of this study did not agree for their data to be shared publicly.

## Appendix A

**Table A1:** Principal component analysis.

	Eigenvalue	% of Variance	Eigenvector
**Principal component 1: “Bacterial tests”**	2.49	24.9	
Number of bacterial tests			0.52
Blood culture collected prior to broad spectrum antibiotic therapy			0.49
Number of CD detected test			0.42
**Principal component** **2: “Operations”**	1.47	14.8	
Rate of surgeries			0.53
Region			−0.58
Average length of stay			0.44
**Principal component 3: “Ability of ICT”**	1.29	12.9	
TDM implementation rate for vancomycin			0.41
Medical fee for IPC type 1			0.60
Contamination of blood cultures			−0.4
**Principal component 4: “Skill in performing blood cultures”**	1.08	10.8	
Multiple sets of blood culture			−0.7
Contamination of blood cultures			0.51

CD, *Clostridioides difficile*; ICT, Infection Control Team; TDM, Therapeutic Drug Monitoring; IPC, Infection Prevention and Control.

**Table A2:** Hospital characteristics according to the five clusters identified using principal component analysis-based cluster analysis: median (interquartile range), or %.

	Cluster 1 (*n* = 25)	Cluster 2 (*n* = 13)	Cluster 3 (*n* = 5)	Cluster 4 (*n* = 49)	Cluster 5 (*n* = 31)	Overall *p*-Value ^g^
**Structure factors**						
Number of beds (beds)	269 (190–490)	275 (218–559)	106 (100–163) ^e^	304 (210–377) ^e^	466 (337–505) ^c,d^	0.0002
Number of patients admissions per year (patients)	6303 (4291–11901)	5789 (3952–12,996) ^c^	2587 (1919–2962) ^b,d,e^	6184 (4429–9231) ^c^	11,046 (8988–13,612) ^c,d^	<0.001
Average length of stay (days)	12.5 (11.2–13.4)	13.4 (10.7–14.8)	14.9 (13.2–20.4) ^e^	13.2 (11.7–15) ^e^	10.9 (9.9–11.8) ^c,d^	<0.001
Rate of surgeries (%) ^f^	22.1 ± 4.8 ^d^	25.3 ± 4.7	29.1 ± 6.3	29.3 ± 5.4 ^a,e^	25.3 ± 3.7 ^d^	0.0055 ^g^
ICU patient admissions (%)	3.0 (0–9.5)	0 (0–5.5)	0 (0–0)	0 (0–4.6)	3.7 (0–4.9)	0.0365
CVC use patients (%)	6.1 (4.7–9.6)	8.0 (5.0–9.7) ^c^	3.1 (1.7–4.7) ^b,e^	5.6 (3.9–8.0)	6.4 (4.7–8.1) ^c^	0.0159
UC use patients (%)	13.3 (9.2–18.7)	12.3 (9.4–14.4)	14.2 (11.2–21.6)	12.1 (9.8–13.4)	12.0 (8.6–15.1)	0.3219
Region (East Japan: %)	92.0	84.6	0	36.7	70.9	
Medical fee for IPC type 1 (%)	84.0	100	0	100	100	
Teaching Hospital (%)	76.0	92.3	60.0	87.8	96.7	
7:1 hospital charge index (%)	92.0	100	100	94.0	100	
Pharmaceutical service (%)	64.0	76.9	40.0	65.3	64.5	
**Process factors**						
TDM implementation rate for vancomycin (%)	64.3 (40.3–73.0) ^b,d,e^	82.1 (60.5–86.8) ^a^	9.7 (4.9–61.5) ^d,e^	80.0 (71.4–87.3) ^a,c^	80.8 (76.3–88.7) ^a,c^	<0.001
Multiple sets of blood cultures (%)	80.6 (66.5–86.5) ^b^	46.3 (13.7–51.5) ^a,c,d,e^	78.0 (74.5–88.5) ^b^	82.3 (75.2–87.3) ^b^	85.0 (74.0–92.4) ^b^	<0.001
Contamination of blood cultures (%)	4.0 (2.5–4.9)	4.1 (1.3–9.2)	3.5 (2.6–5.3)	3.0 (1.8–4.2)	2.4 (1.9–3.7)	0.0988
Number of CD detected test (/1000 bed-days)	3.8 (1.5–5.1)	3.7 (2.5–4.9)	4.4 (1.6–8.2)	3.8 (2.5–4.6) ^e^	5.4 (4–6.1) ^d^	0.0114
Blood culture collected prior to broad spectrum antibiotic therapy (%)	40.6 (34.8–53.6) ^d,e^	60.9 (37.7–74.8)	30.1 (21.4–45.1) ^e^	59.2 (41.8–64.6) ^a,e^	72.3 (66.3–79.6) ^a,c,d^	<0.001
Specimens for culture prior to broad spectrum antibiotic therapy (%)	72.8 (63.4–80.4) ^d,e^	87.3 (72.3–90.2)	63.0 (43.9–68.4) ^e^	81.8 (74.6–85.7) ^a,e^	85.9 (83.5–92.3) ^a,c,d^	<0.001
Number of bacterial tests (/100 bed-days)	6.8 (5.6–9.4) ^e^	10.3 (7.2–11.1) ^e^	6.7 (2.4–9.3) ^e^	8.5 (6.5–10.5) ^e^	14.6 (11.6–19.0) ^a,b,c,d^	<0.001
AUD of antibiotic injection (/100 bed-days)	15.4 (12.1–17.4) ^e^	18.3 (14.1–20.2)	12.8 (10.5–16.7)	14.5 (11.1–16.0) ^e^	19.1 (17.2–21.9) ^a,d^	<0.001
DOT of antibiotic injection (/100 bed-days)	25.6 (21.7–28.4) ^e^	28.3 (26.1–31.4) ^d^	24.9 (16.7–26.3)	23.5 (19.6–26.8) ^b,e^	30.1 (26.6–32.3) ^a,d^	<0.001

ICU, Intensive care unit; CVC, Central venous catheter; UC, Urinary catheter; IPC, Infection Prevention and Control; TDM, Therapeutic Drug Monitoring; CD, *Clostridioides difficile*; AUD, Antimicrobial Use Density; DOT, Days of Therapy; MRSA, Methicillin-resistant *Staphylococcus aureus*. ^a^ different form Cluster 1 (*p* < 0.05), ^b^ different form Cluster 2 (*p* < 0.05), ^c^ different form Cluster 3 (*p* < 0.05), ^d^ different form Cluster 4 (*p* < 0.05), ^e^ different form Cluster 5 (*p* < 0.05). ^f^ mean ± SD. ^g^ ANOVA/Tukey–Kramer test.

**Table A3:** Definition of variables.

Name	Units	Definition
**Structure factors**		
Number of beds	Beds	Number of hospital beds
Number of admissions patients per year	Patients	
Average length of stay	Days	
Rate of surgeries	%	Number of JANIS SSI surveillance/Number of all surgeries × 100
ICU admission patients	%	ICU admission patients/Number of admission patients per year
CVC patients	%	Number of Central Venous Catheter used patients/Number of admission patients per year × 100
UC patients	%	Number of urinary Catheter used patients/Number of admission patients per year × 100
Region	%	West Japan = 0, East Japan = 1
Medical fee for IPC type 1	%	Medical fee for IPC type 2 = 0, Medical fee for IPC type 1 = 1
Teaching Hospital	%	Non-Teaching Hospital = 0, Teaching Hospital = 1
7:1 hospital charge index	%	10:1 hospital charge index = 0, 7:1 hospital charge index = 1
Pharmaceutical service	%	Non-Pharmaceutical service = 0, Pharmaceutical service = 1
**Process factors**		
TDM implementation rate for vancomycin	%	TDM performed patients in denominator/Patient treatment duration >3 days for vancomycin
Multiple sets of blood culture	%	Number of patients in whom multiple blood cultures were taken/Total number of patients who blood cultures were taken
Contamination of blood cultures	%	Number of contaminated cultures/Number of patients in whom multiple blood cultures were taken
Number of CD detected tests	/1000 bed-days	Number of CD detected tests/length of hospital stay for inpatients × 1000
Blood culture collected prior to broad spectrum antibiotic therapy	%	Before starting broad spectrum systemic antibiotic therapy in hospitalized adults with at least one blood culture/Admitted broad spectrum systemic antibiotic therapy
Specimens for culture prior to broad spectrum antibiotic therapy	%	Before starting broad spectrum systemic antibiotic therapy in hospitalized adults with bacterial culture/Admitted broad spectrum systemic antibiotic therapy
Number of bacterial tests	/100 bed-days	Number of bacterial tests/length of hospital stay for inpatients × 100
AUD of antibiotic injection	/100 bed-days	Antimicrobial consumptions (g)/(DDD ^a^ × length of hospital stay for inpatients) × 100
DOT of antibiotic injection	/100 bed-days	DOT/length of hospital stay for inpatients × 100
**Antimicrobial resistance**		
MRSA/*S. aureus* detection rate	%	MRSA detected patients/Number of MRSA + MSSA detected patients × 100

^a^ Used WHO ATC/DDD index ver.2016. JANIS: Japan Nosocomial Infections Surveillance; SSI: Surgical site infection ICU, Intensive care unit; CVC, Central venous catheter; UC, Urinary catheter; IPC, Infection Prevention and Control; TDM, Therapeutic Drug Monitoring; CD, *Clostridioides difficile*; AUD, Antimicrobial Use Density; DOT, Days of Therapy; *S. aureus, Staphylococcus aureus*; MRSA, Methicillin-resistant *S. aureus*; MSSA, Methicillin-susceptible *S. aureus*.
